# High-Performance Flexible Sensor with Sensitive Strain/Magnetic Dual-Mode Sensing Characteristics Based on Sodium Alginate and Carboxymethyl Cellulose

**DOI:** 10.3390/gels10090555

**Published:** 2024-08-27

**Authors:** Chong Liu, Longwang Yue, Yu Fu, Zhenshuai Wan, Li Wang, Yangke Wei, Sha Li

**Affiliations:** 1 School of Mechanical and Electrical Engineering, Henan University of Technology, Zhengzhou 450001, China; 2 School of Mechanics and Safety Engineering, Zhengzhou University, Zhengzhou 450001, China

**Keywords:** flexible electronics, wearable electronics, SA/CMC cellulose porous sponges, double network, crosslinking, responsiveness, multisensing modes, gel synthesis

## Abstract

Flexible sensors can measure various stimuli owing to their exceptional flexibility, stretchability, and electrical properties. However, the integration of multiple stimuli into a single sensor for measurement is challenging. To address this issue, the sensor developed in this study utilizes the natural biopolymers sodium alginate and carboxymethyl cellulose to construct a dual interpenetrating network, This results in a flexible porous sponge that exhibits a dual-modal response to strain and magnetic stimulation. The dual-mode flexible sensor achieved a maximum tensile strength of 429 kPa and elongation at break of 24.7%. It also exhibited rapid response times and reliable stability under both strain and magnetic stimuli. The porous foam sensor is intended for use as a wearable electronic device for monitoring joint movements of the body. It provides a swift and stable sensing response to mechanical stimuli arising from joint activities, such as stretching, compression, and bending. Furthermore, the sensor generates opposing response signals to strain and magnetic stimulation, enabling real-time decoupling of different stimuli. This study employed a simple and environmentally friendly manufacturing method for the dual-modal flexible sensor. Because of its remarkable performance, it has significant potential for application in smart wearable electronics and artificial electroskins.

## 1. Introduction

Flexible sensors can recognize, respond to, and monitor various stimuli, including pressure [1,2,3], strain [4,5,6], temperature [7,8,9], humidity [10,11,12], and gas [13,14,15,16]. Their integration into wearable electronic devices has attracted increasing attention. The fundamental principle underlying flexible sensor research is the conversion of external mechanical stimuli into electrical signals that are analogous to the function of biological skin [17,18,19]. Furthermore, compared with other sensing modalities, magnetic field excitation enables contactless sensing and offers superior response speed and environmental adaptability [20]. Nevertheless, flexible sensors that are limited to the detection of a single type of stimulus are inadequate in some cases, and it is important to develop flexible sensors that can simultaneously detect multiple stimuli is important. In addition, multifunctional sensors that incorporate both strain and magnetic sensing capabilities have promising applications in remote human–computer interactions [21,22], wearable electronics [23,24], and soft robotics [25].

Magnetic particles (MPs) can be integrated into the sensor substrates to create highly promising magnetorheological materials because of their rapid reversibility and adjustable mechanical properties [26,27,28]. Therefore, these materials are suitable for use in composite applications. In our previous studies, multiwalled carbon nanotubes (MWCNTs), gelatin (GE), nanoscale Fe_3_O_4_, and micron-sized carbonyl iron particles (CIPs) were used as electrochemical materials via surface modification techniques [29,30,31]. Additionally, magnetically active materials in the form of bi-disperse MPs with core–shell structures have been synthesized using the sol–gel method [32,33]. Nanomagnetic particles coated with MWCNTs and GE exhibited excellent dispersion stability, electrical conductivity, and magnetic sensitivity. Notably, the inclusion of MWCNTs enhanced the conductivity of the matrix. These properties render them highly favorable as active fillers for dual-mode sensors.

The selection of the flexible sensor substrate significantly affects the performance of the sensor. A well-designed matrix facilitates the dispersion of electroactive materials, thereby minimizing the risks of oxidation and corrosion. When electroactive materials are uniformly dispersed, they respond rapidly to external stimuli. Various materials, such as gels [34], elastomers [35], plastomers [36], sandwich structures [37], fibers [38], and porous substrates [39,40], have been employed in the development of flexible sensors. The effective dispersion and deformation capabilities of the active materials within the matrix play a crucial roles in determining sensor performance. Notably, the internal pores of the porous structure are uniformly distributed with adjustable pore diameters, contributing to the overall flexibility and reliability of the substrate and making it an ideal choice for flexible sensor applications, such as silicone rubber [41], polyurethane [42], natural biopolymers [43], and polydimethylsiloxane [44]. However, owing to their inherent characteristics, synthetic materials still have major problems in terms of flexibility and repeatable. Compared to other traditional porous-structured polymer substrates, natural biopolymers are chemically stable owing to their diverse sources and complex molecular structures and easily improved performance through modification, and they are a green materials [45]. Among natural biopolymers, sodium alginate (SA) and carboxymethyl cellulose (CMC) offer distinct advantages over other porous structure polymer substrates, such as complete biodegradability, low cost, structural diversity, and strong sensitivity [46,47]. These high-molecular-weight natural polymers possess numerous functional groups that enable the use of electrostatic interactions to form physically crosslinked networks within porous polymer substrates [39]. This approach significantly enhances the mechanical strength and structural stability of flexible sensor substrates.

SA is an abundant natural polymer material that is abundantly found in nature [48]. Its mechanical properties can be enhanced via crosslinking with Ca^2+^ [49]. Zhang et al. used guar gum (GG) to improve the toughness of SA and applied it in wearable devices, where the maximum stress reached 36.5 kPa and the maximum strain was 7.3% [50]. CMC is characterized by its long-chain polysaccharide structure [51], and its inherently high porosity makes it a suitable particularly suitable as a substrate for porous structures [52]. However, CMC alone is extremely brittle and exhibits poor resilience. The abundant carboxyl groups in CMC are typically crosslinked to enhance its elasticity and flexibility [51]. Qin et al. developed a strain hydrogel sensor with good mechanical strength using double-network crosslinking between GE and CMC [53]. In this study, CMC was used to enhance the porous structure of SA. Using the hydrogen bonding interactions between SA and CMC macromolecules, along with the chelation reaction of SA with Ca^2+^, a dual crosslinked network was established to address the material’s low mechanical strength of the material. The resulting product exhibited excellent mechanical strength and sensing properties, confirming its potential application as a flexible wearable sensor.

In this study, we developed a porous flexible sponge sensor that exhibited a dual-mode response to strain and magnetic stimuli. The sensor was fabricated using SA and CMC as the primary materials, and Ca^2+^ as the crosslinking agent. Additionally, we incorporated core–shell bi-disperse MPs for physical reinforcement by employing an in situ freeze-polymerization process. Investigation of the electrical response and mechanical properties of the sensor under various external stimuli reveals that the SA/CMC flexible sensor demonstrated excellentl mechanical properties. The sensor exhibited rapid response times and significant signal changes when subjected to external magnetic and mechanical stimuli. Furthermore, its swift strain response and increased sensitivity to magnetic fields render the SA/CMC flexible sensor suitable for use as a wearable motion sensor capable of monitoring human joint movements (such as those of the fingers and wrists) or detecting the distribution of external pressure and magnetic fields. Consequently, our research offers a promising solution for the advancement of next-generation soft robotics and wearable electronics.

## 2. Results and Discussion

### 2.1. SA/CMC Porous Sponge Formation

**Figure 1:**
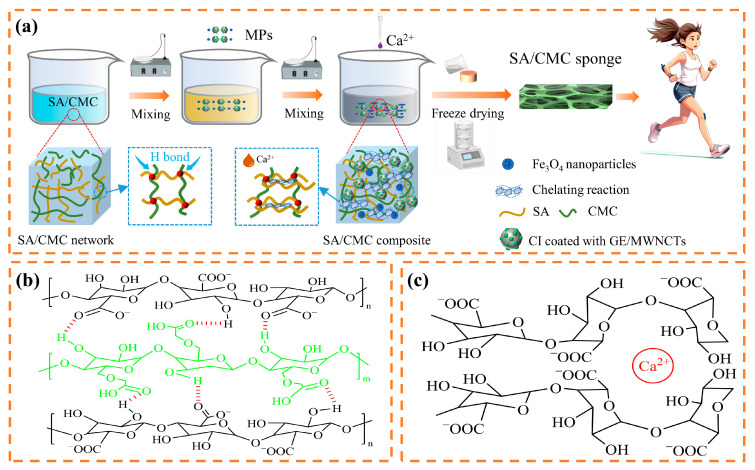
(**a**) Schematic of the fabrication process for SA/CMC porous sponges. Principles of (**b**) biological crosslinking reaction and (**c**) chelation reaction.

The preparation method for the SA/CMC porous sponge sensor, which exhibited strain and magnetic dual-modal responses, is illustrated in Figure 1a. First, SA and CMC were dissolved in distilled water and mixed thoroughly. SA macromolecules, which are abundant in carboxyl and hydroxyl groups, formed hydrogen bonds with the carboxymethyl groups present in the CMC macromolecules. This interaction resulted in the entanglement of the two polymers and leads to the formation of an initial crosslinked network (Figure 1b). Concurrently, the spatial volume effect of the –CH_2_OCH_2_COO groups within the CMC molecular structure enhanced the internal porosity of the resulting complex. Subsequently, bidispersed MPs were introduced and mixed, after which a solution of CaCl_2_ and glycerol (GL) was added. The multiple oxygen atoms on SA facilitated the chelation with Ca^2+^, thereby tightening the binding of the SA chains, as shown in Figure 1c. The composite was subjected to ultrasonic oscillation and vacuum drying to gradually remove the entrapped bubbles. Finally, the composite gel was frozen and vacuum-dried to yield a lightweight, dry SA/CMC porous sponge composite. The formation of hydrogen and ionic bonds by physical crosslinking contributes to the establishment of a double-crosslinked network within the composite, which enhances its mechanical properties and stability. The synthesized porous sponge composites were subjected to a series of characterizations and performance evaluations.

### 2.2. Material Characterization of the SA/CMC Porous Sponge

**Figure 2:**
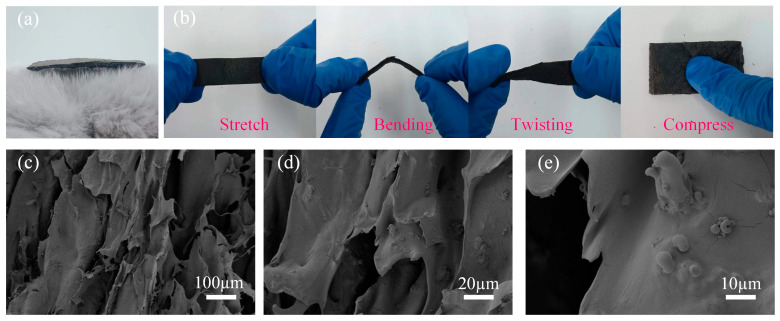
(**a**) S4 sponge placed on plush. (**b**) Deformation test on S4 sponge. (**c**–**e**) SEM images of S4 sponge at different magnifications.

All the prepared complexes are shown in Figure 2a. The SA/CMC porous sponge, measuring 20 mm × 10 mm × 5 mm, was positioned on the plush surface without causing any indentation because of its low density. The complexes were subjected to various mechanical tests, including stretching, bending, twisting, and pressing, to demonstrate their deformation capacities. This indicates that the SA/CMC porous sponge complexes possessed excellent flexibility (Figure 2b). Figure 2c–e show the microstructure of sample S4 observed using a scanning electron microscope (SEM). The results indicate that the stomata were uniformly distributed throughout the complexes, with micrometer-sized CIPs and nanometer-sized Fe_3_O_4_ evenly dispersed within the stomata.

**Figure 3:**
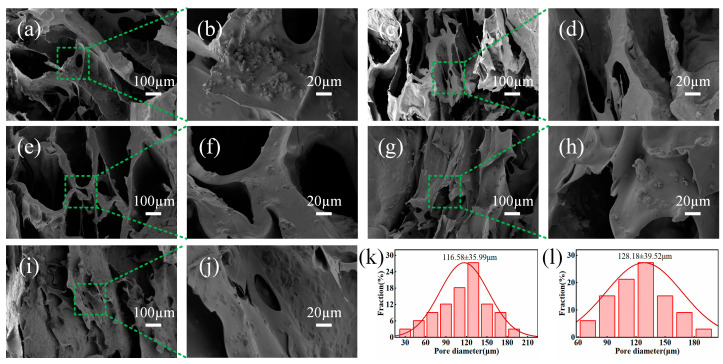
Microstructure images of SA/CMC samples at different magnifications of (**a**,**b**) S1, (**c**,**d**) S2, (**e**,**f**) S3, (**g**,**h**) S4, (**i**,**j**) S5, (**k**) pore diameters of S4, (**l**) pore diameters of S3.

The microstructures of the SA/CMC porous sponge complexes with various SA/CMC mass ratios are shown in Figure 3. All samples exhibited a significant presence of MPs and pores of varying diameters. As the SA content increased, both the pore diameter and pore wall thickness increased gradually, whereas the number of pores decreased. Conversely, a reduction in the CMC content led to a decrease in both the pore wall thickness and pore diameter. Notably, higher concentrations of the SA/CMC complexes demonstrated improved densification and homogeneity, which were attributed to enhanced electrostatic interactions. S4 exhibited the most uniform pore structure and size (Figure 3k). In contrast, the CMC molecular structure contained a large amount of -CH_2_OCH_2_COO-, and with an increase in the content, the spatial volume effect was enhanced, which resulted in thickening of the pore wall and enlargement of the pores. S3 displayed larger pore walls and reduced flexibility owing to its higher content (Figure 3l). Based on these observations, the S4 porous sponge complex was selected for further investigation.

**Figure 4:**
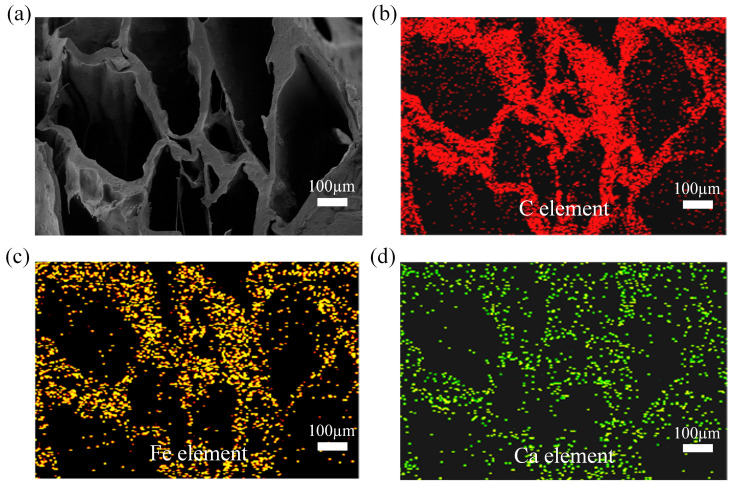
(**a**) SEM image of the sponge sample. (**b**–**d**) Elemental mapping images of sponge samples.

The distributions of C, Fe, and Ca within the complexes were analyzed using energy-dispersive spectroscopy (EDS), as illustrated in Figure 4. The results indicate that elemental C was uniformly distributed throughout the SA/CMC complexes (Figure 4b). Similarly, Fe exhibited a uniform distribution within the complex (Figure 4c), suggesting that the MPs were effectively integrated within the complex, enabling the SA/CMC sponge complex to respond to an external magnetic field. Furthermore, Ca was uniformly dispersed within the sponge complex skeleton (Figure 4d), which is attributed to the chelation reaction between SA and Ca^2+^ (Figure 1c). This interaction results in crosslinking and entanglement of the molecular chains, forming a complex with an eggshell structure. Consequently, magnetic SA/CMC porous sponge complexes featuring a three-dimensional porous network were successfully synthesized, which is consistent with the SEM results (Figure 4a).

**Figure 5:**
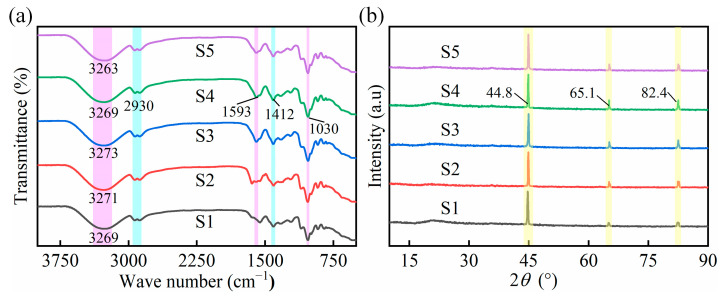
(**a**) FTIR spectra of various SA/CMC porous sponges. (**b**) XRD pattern of various SA/CMC porous sponges.

Fourier Transform Infrared (FTIR) and X-ray Diffraction (XRD) analyses confirmed the structure and properties of the SA/CMC complexes. Figure 5a shows the FTIR spectra of the porous sponge complexes of samples S1–S5 in the range of 4000–500 cm^−1^ range. By comparing the five sets of samples, it was found that they migrated continuously in the O–H vibrational peaks at 3400~3200 cm^−1^, indicating that SA interacted with CMC and that the hydrogen bonds formed between the polymer and water molecules were changed. Taking the S4 sample as an example, the asymmetric and symmetric stretching vibrations of –COO- were observed at 1593 and 1412 cm^−1^, respectively. The variation in the vibrational absorption peak at 1593 cm^−1^ was caused by the ionic crosslinking reaction of SA with Ca^2+^ and the electrostatic interaction of SA with CMC. The band located at 1030 cm^−1^ is the absorption peak of C–O, and the band located at 2930 cm^−1^ is the stretching vibration of –CH_2_–. The characteristic peak at 3279 cm^−1^ is related to the vibration of O-H, indicating the formation of hydrogen bonds between the polymer and water molecules. The O-H characteristic peaks become wider and stronger, from 3400 to 3200 cm^−1^, from samples S1 to S3 and S5 to S3, which suggests that the hydrogen bonding is enhanced with the increase in SA and CMC content. The XRD patterns of the SA/CMC samples are shown in Figure 5b. The characteristic peaks near 44.8°, 65.1° and 82.4° correspond to the (100), (200) and (211) reflection planes of the MPs, respectively. These characteristic peaks were the same as those of the MPs reported in the literature [32], indicating that the added MPs were not affected by other substances. It was also found by comparing the five samples that all the characteristic peaks were consistent; therefore, the content of SA and CMC had no effect on the crystalline structure.

**Figure 6:**
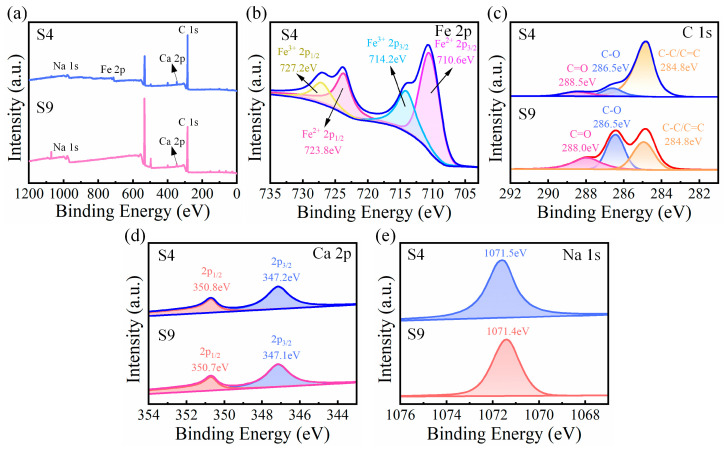
(**a**) XPS survey spectra of S4 and S9. (**b**) Fe 2p, (**c**) C 1s, (**d**) Ca 2p, and (**e**) Na 1s spectra.

The X-ray photoelectron spectroscopy (XPS) plots of the major elements (Na, Fe, Ca, and C) in the SA/CMC porous sponge complexes are shown in Figure 6a, and the Fe 2p spectrum of the S4 sample proved that the SA/CMC hybrid complexes were successfully doped with MPs (Figure 6b). In the Fe 2p spectrum of S4, the Fe 2p_3/2_ (710.6 eV) and Fe 2p_1/2_ (723.8 eV) peaks corresponded to Fe^2+^. The Fe 2p_3/2_ (712.4 eV) and Fe 2p_1/2_ (726.1 eV) peaks are assigned to Fe^3+^ (Figure 6b). For the C 1S spectrum, three peaks, 284.8, 286.5, and 288.0 eV, corresponding to C–C/C=C bonds, C–O bonds, and C=O bonds, respectively, were decomposed by fitting in the S9 sample (Figure 6c). In contrast to the S4 sample, a shift in the C=O binding energy (288.5 eV) was observed after sdding MPs, indicating that the carboxyl group in SA coordinated with the metal ion. In Figure 6d, the Ca 2p spectra of the S9 porous complexes correspond to Ca 2p_3/2_ and Ca 2p_1/2_ at 347.1 and 350.7 eV, respectively. Comparing the S4 sample, it can be found that the binding energies of Ca 2p_3/2_ and Ca 2p_1/2_ are positively shifted slightly to 347.2 and 350.8 eV upon the addition of MPs. Additionally, the binding energies in the Na 1s were observed to be shifted from 1071.4 to 1071.5 eV, with a positive shift of 0.1 eV (Figure 6e). By comparing samples S4 and S9, it was found that the binding energies of the elements shifted within a certain range after the addition of MPs, which may have been caused by the electrostatic interactions of the MPs with the substrate inside the complex.

**Figure 7:**
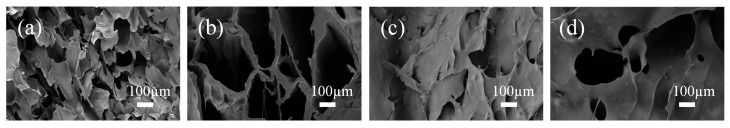
Microstructure images of different SA/CMC samples. (**a**) S6. (**b**) S4. (**c**) S7. (**d**) S8.

Figure 7 shows the microstructural morphologies of the complexes after treatment with different GL concentrations. The results show that the GL content significantly affected on the microstructure of the porous complexes. When the GL concentration was increased from 0 to 0.8 wt%, the thickness of the pore wall decreased, and the porosity increased; GL made the porous structure of the complexes more homogeneous and dense owing to its good water-holding capacity. GL acts as a humectant and plasticizer, squeezing the backbone structure by isolating the macromolecules in the complex and allowing water to swell sufficiently during the freezing process, thereby making the complex more porous after drying. In contrast, GL acts as a lubricant between the chains of macromolecules, allowing the molecules to rotate and slip into each other internally, thereby increasing the toughness of the porous structure and causing a less brittle fracture of the matrix skeleton. However, at higher GL contents (4–8 wt%), excessive porosity reduced the stability of the backbone structure.

**Figure 8:**
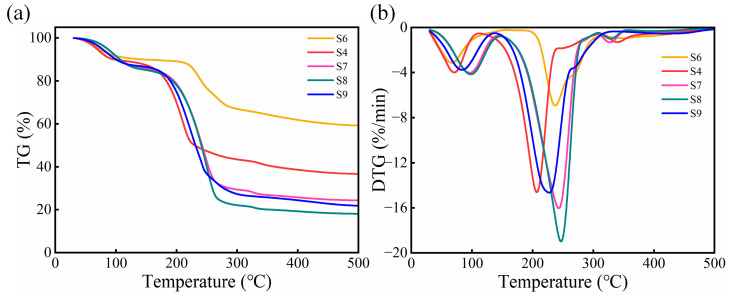
(**a**,**b**) TG and DTG curves of SA/CMC samples with different glycerol content.

The effects of GL and MPs on the thermal stabilitiy of the porous sponge complexes were investigated. The thermogravimetric (TG) and derivative thermogravimetric (DTG) curves of the samples are shown in Figure 8. The GL content in samples S6, S4, S7, and S8 were 0, 2, 4 and 8 wt%, respectively. No MPs were added to S9. The thermal degradation process was divided into three steps. First, the S4 sample showed a peak weight loss of 15% at 180 °C, owing to water loss. In the temperature range of 180–250 °C, the weight loss of S4 was 35%, while the weight loss of S6, S7, S8, and S9 samples were 20%, 55%, 65%, and 60%, respectively. This was attributed to the weight loss due to the breakage of the macromolecular chains in the complexes, as well as the combination of adjacent carboxyl and hydroxyl groups to form water molecules, followed by evaporation. Finally, owing to the secondary decomposition of carbon atoms in the SA/CMC complexes, the final weight losses of the five samples remained stable. Thus, GL acts as a small molecule that can be interspersed into complex molecules, altering the interaction forces and chemical bonds between the molecular chains. With increasing temperature, the hydrogen bonds formed between the SA and CMC break or weaken, making it easier for the samples to decompose. In addition, the MPs increased the thermal conductivity of the sample and promoted heat dissipation during heating, thereby reducing the heat accumulation and decomposition rate of the sample.

In summary, the synthesis mechanism of SA/CMC porous sponge complexes was elucidated in Figure 1a. The structure of SA is similar to that of CMC, and the carboxymethyl stretch on the CMC macromolecule is interspersed between the SA macromolecules, which forms hydrogen bonds with the carboxyl and hydroxyl groups on the SA macromolecules and improves the low mechanical strength of ionically crosslinked SA. A strong interaction exists between these two macromolecules, and this interaction is enhanced with an increase in the CMC content, whereas the spatial volume effect of –CH_2_OCH_2_COO– in the molecular structure of CMC can increase the internal pores of the complex. Concurrently, multiple oxygen atoms on SA can chelate with Ca^2+^, which makes the inter-chain bonding of SA tighter and more synergistic. The interactions between the molecular chains ultimately lead to the formation of a double-crosslinked network structure, which not only enhances the connectivity of the internal pores of the porous structure but also optimizes its molecular arrangement. Their synergistic effect is expected to improve the structural stability and load-bearing capacity of the SA/CMC porous sponge complexes. In addition, the MPs were stably bound to the matrix of the SA/CMC porous sponge complexes through electrostatic interactions and uniformly dispersed in the sponge substrate.

### 2.3. Mechanical Properties and Hydrophilicity of the SA/CMC Porous Sponge

**Figure 9:**
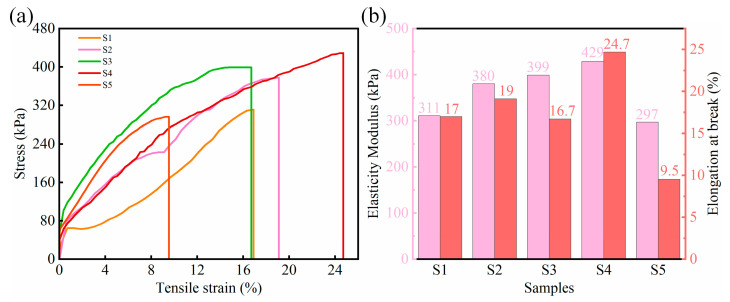
(**a**) Tensile stress–strain curves for different SA/CMC samples. (**b**) Modulus of elasticity and elongation at break of different SA/CMC samples.

Analysis of tensile mechanical properties is necessary to obtain high-performance porous sponges. Figure 9a shows the tensile stress–strain curves of the complex specimens at five SA and CMC mass ratios. The tensile stresses of the complexes are approximately linear at low strain levels during the initial stages. Overall, the complexes exhibited elastic deformation and tensile properties until the final fracture. The elastic moduli and elongations at the break of the complex samples were analyzed and recorded, as shown in Figure 9b. When the SA mass fraction was increased from 1 to 3 wt%, the tensile strength gradually increased from 311 to 429 kPa, primarily because of the increased thickness of the pore walls inside the complex. The elongation at break of the specimen increased with increasing CMC content, and the flexibility improved; however, an excessive amount of CMC also reduced the elongation at break of the specimen. This may be related to the aggregation of the CMC. The S4 sponge had the highest modulus of elasticity (429 kPa) among the samples, whereas its elongation at break under an external force or strain was 24.7%.

**Figure 10:**
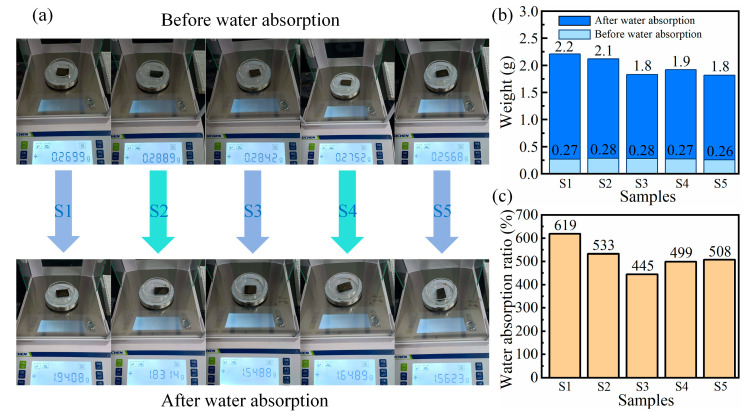
(**a**) Photographs of various SA/CMC samples before and after water absorption. (**b**,**c**) Weight and water absorption ratio *Rw* for various samples, respectively.

The hydrophilicity of the SA/CMC porous sponge complexes also affects their porosity and water absorption capacity. Figure 10a shows the digital images from the water absorption tests of the complex samples with varying mass fractions at the initial and stabilization stages. The results for the corresponding samples are shown in Figure 10b,c. The water absorption of the SA/CMC complexes gradually decreased with increasing SA content. When the SA content was high, ionic crosslinking with Ca^2+^ was enhanced, leading to a denser three-dimensional network that made it more difficult for water molecules to enter the pores. With increasing SA content, the pore diameter increased, the thickness of the pore wall increased gradually, and the number of pores decreased. Minimum water absorption occurred at an SA content of 3 wt%. Notably, the water absorptions values of samples S1, S2, S3, S4 and S5 were 619%, 533%, 445%, 499% and 508% of their own weight, respectively. Excessive CMC content caused the complexes to form more hydrogen bonds, which, in turn, affected the penetration of water molecules. In addition, the freeze-drying technology used for the complexes favored the production of a uniform and wide pore structure in the film. This is conducive to the full diffusion of water molecules into the pores and improves water absorption. In summary, S4 exhibited good flexibility, deformability, and high mechanical strength.

### 2.4. Strain and Magnetic Sensing Properties of SA/CMC Porous Sponge Sensors and Human Motion Detection Applications

**Figure 11:**
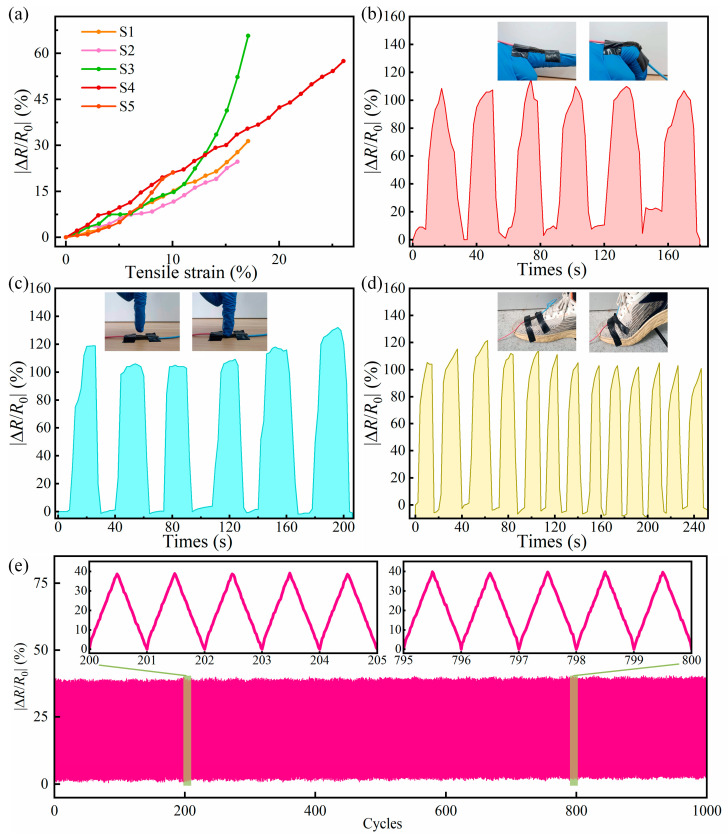
Response of flexible sensor S4 to strain stimuli. (**a**) Relative resistance variation as a function of tensile strain for different SA/CMC sponges. Photographs and relative resistance variation, along with the time of the S4 sponge sensor excited by (**b**) finger bending, (**c**) finger compression, and (**d**) vamp bending. (**e**) Long-term durability evaluation of the SA/CMC sensor under 1000 loading–unloading cycles at 20° bending strain.

The SA/CMC porous sponge complexes incorporated MPs and MWCNTs in a dispersed state to form an internal conductive network, enabling the complexes to electrically respond to strain stimuli and become sensitive contact strain sensors by applying different external stimuli. Consequently, it was necessary to evaluate the mechanical stimuli responsiveness of the complexes. We systematically investigated the sensing properties of the SA/CMC porous sponge complexes under various deformation conditions, such as stretching, bending, and compression. Figure 11a shows the tensile strain of various SA/CMC porous sponge complexes as a function of $\left|∆R/R_{0}\right|$. When the complexes were stretched, their $\left|∆R/R_{0}\right|$ increased accordingly, varying from 0% to 57.5%. Upon comparing the five samples, it was observed that the maximum $\left|∆R/R_{0}\right|$ of samples S1, S2, S3, S4, and S5 were 31.4%, 24.7%, 52.3%, 57.5%, and 21.2%, respectively. Notably, the S4 porous sponge complex exhibited better tensile responsiveness, which not only had the maximum $\left|∆R/R_{0}\right|$ but also the maximum tensile rate. Therefore, the S4 porous sponge complex was selected for further analysis.

Owing to the excellent flexibility of the porous sponge complex, it was attached to the back of a finger for the bending strain response test, as shown in Figure 11b. The $\left|∆R/R_{0}\right|$ changed significantly when the finger was bent and can change up to 115% at 90°, which indicates that it senses bending well and recovers effectively.. Repeating the bending action of the finger can yield an electrical response using the same change rule. The results of the electrical response to compressive stress stimulation are shown in Figure 11c. When a finger was pressed on the S4 porous sponge complex, the complex was observed to change immediately and synchronously and then return to its original state, and the same change in $\left|∆R/R_{0}\right|$ occurred after repeatedly pressing the finger. This indicates that the complex has significant response sensitivity and reliable stability. The complex could also be mounted on the surface of a shoe and used to detect human walking data, as shown in Figure 11d. When walking, the surface of the shoe bends and the complex quickly generated an electrical response, and $\left|∆R/R_{0}\right|$ changed up to 105% when the pressure was maximized. The electrical response of the complex quickly disappeared after lifting the foot, and an electrical response with the same trend was obtained by repeating the walking action multiple times. The long-term durability evaluation of the SA/CMC sensor under 1000 loading–unloading cycles at a bending strain of 20° is shown in Figure 11e. The SA/CMC porous sponge complex exhibits excellent strain response and superior stability. Therefore, the SA/CMC porous sponge complexes have great application prospects for the real-time detection of human joint motion.

**Figure 12:**
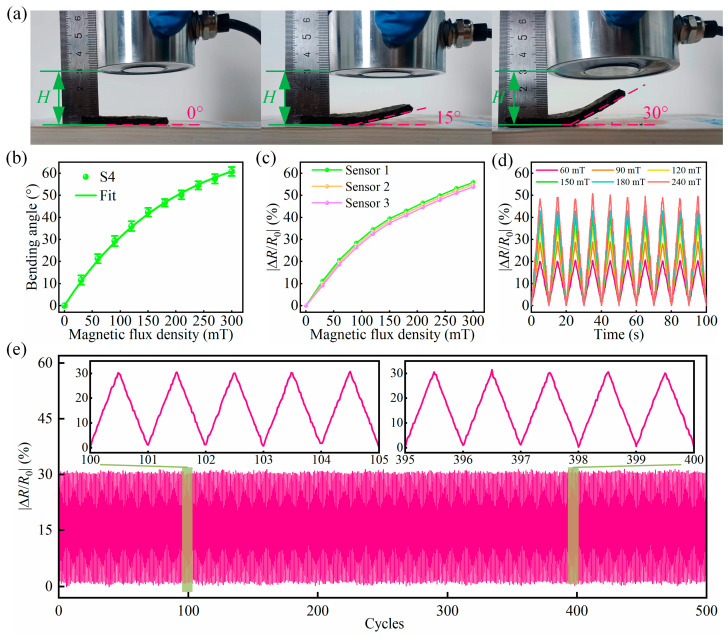
Response of the flexible sensor S4 to a magnetic field. (**a**) Bending angle of the S4 sensor under different magnetic fields. (**b**) Bending angle–magnetic field curves of the S4 sensor. (**c**) Relative resistance–magnetic field curves of three different S4 sensors. (**d**) $|∆R/R_{0}|\;$ of S4 sensors under different magnetic fields for 10 cycles, and (**e**) long-term durability evaluation of the SA/CMC sensor during 500 cycles under 100 mT.

After incorporation of bidispersed MPs into the SA/CMC porous sponge complex, it was necessary to investigate their magnetic response properties. To verify whether the SA/CMC complex could produce a physical response after encountering a magnetic field, an applied magnetic field force was applied and withdraw from the sample. When it was applied and withdrawn, as shown in Figure 12a, it was observed that changing the intensity of the electromagnetic field allowed the sample to be bent at different angles. Because the particles between the MPs doped into the SA/CMC sponge interact with each other under a magnetic field and bend, they can be used as lightweight magnetic field sensors to detect magnetic fields.

The S4 sensor was investigated in detail. By applying a magnetic field of gradually increasing strength to the S4 sensor, the bending angle and resistance change were observed to gradually increase (Figure 12b,c), thus proving that the sensor possessed excellent deformation capability and magnetic sensitivity. A cyclic magnetic field was applied to the S4 sensor, and the peak value of $\left|∆R/R_{0}\right|$ of the sample increased significantly from 20.1% to 28.7%, 35.2%, 39.8%, 42.8%, and 50.2% under different magnetic field strengths. This demonstrates that the magnetic response performance of the sensor has good reproducibility (Figure 12d) and a stable electrical response (Figure 12e). For the above phenomenon, it can be assumed that the bi-dispersed MPs within the substrate become dense under the action of an external magnetic field, which affects the formation of internal conductive paths and leads to a change in the resistance.

Table 1 presents a comparison of the functionality and performance of the SA/CMC dual-mode flexible sensors and previously reported samples. The comparison shows that our dual-mode sensor exhibits competitive advantages for strain and magnetic field stimuli. In summary, the prepared SA/CMC porous sponge sensors exhibited strain/magnetic bimodal sensing performance and were capable of excellent electrical responsiveness and superior stability under tensile, bending, and compressive strains, as well as under magnetic field stimulation, which is promising for application in human wearable electronics, flexible robotics, and human–machine interaction.

### 2.5. Stimulus–Response Mechanism of SA/CMC Porous Sponge

**Figure 13:**
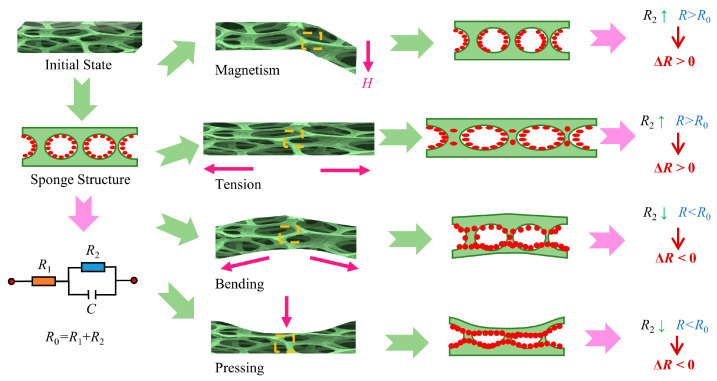
Sensing mechanisms of SA/CMC porous sponge sensors under external magnetic field, tension, bending, and compression stimuli.

In this study, the bimodal stimulus–response mechanism of the SA/CMC porous sponges mainly relied on the contact area and dispersion characteristics of the conductive MWCNT network and bidispersed MPs within the microstructure. As shown in Figure 13, the conductivity of the sensor can be divided into two parts: the inherent conductivity resistance *R*_1_ of the conductive particles themselves and the conductivity between the conductive particles, represented by resistance *R*_2_ and capacitance C in parallel. In particular, the total resistance can be expressed as *R*_1_ + *R*_2_ at DC voltage, where resistance *R*_1_ is a constant value determined by the properties of the MPs themselves. *R*_2_ is determined by the distance between neighboring conductive particles in the sponge substrate network. To further explore the stimulus–response mechanism, responses to various external stimuli are shown in Figure 13.

At rest, the MPs were randomly dispersed on the sponge skeleton. When a magnetic field was applied, magnetic stresses were generated among the MPs, which aggregated along the direction of the magnetic susceptibility lines and led to deformation of the porous sponge. The MPs inside the sponge substrate were arranged in chains along the magnetic susceptibility lines. The originally disorganized conductive particles increased their distance perpendicular to the direction of the magnetic field lines and were isolated from each other. Thus, the resistance output, *R*_2_, increased. After the disappearance of the magnetic field, the conductive paths were restored, and the resistance returned to its original value. Similarly, after the application of tensile strain, the distance between the conductive particles inside the sponge substrate gradually increased; thus, the resistance *R*_2_ increased. In contrast, when the sensor was subjected to external bending strain, the upper and lower sides of the porous sponge were deformed in opposite directions, particularly in the compression layer, resulting in smaller pores. This reduced the contact distance of the conductive particles in the compression layer, making it easier to generate a conductive pathway, thus leading to a decrease in resistance *R*_2_. Similarly, when the sensor was compressed, the longitudinal distance of the sponge pores decreased, and the conductive particles were compressed against each other, thereby making it easier to generate conductive pathways, which resulted in a decrease in resistance *R*_2_. Thus, the SA/CMC porous sponge sensor not only detects strain and magnetic stimuli but also distinguishes these stimuli based on the changing characteristics of the signals.

### 2.6. Limitations and Future Work

This study had some limitations. For example, the samples used in this study were relatively small and could not be used with a large load-carrying capacity. The effect of the porous sponge sensor on the sensing performance in environments with different humidities remains unclear. Further research is required to overcome these limitations. In addition, it is worthwhile to pay special attention to the application expansion of flexible sensors, especially their application in the perception of soft robots and human–computer interaction based on machine learning.

## 3. Conclusions

In this study, a high-performance dual-modal SA/CMC sensor was developed by incorporating GE/MWCNTs and bidispersing MPs into SA/CMC porous sponge complexes. The sensor operates based on the principle of constructing an ionic crosslinked network between SA and metal ions, coupled with the introduction of CMC, to establish a secondary hydrogen bond network between SA and CMC, thereby forming a double-network interpenetrating structure. The SA/CMC porous sponge composite exhibited excellent mechanical strength and hydrophilicity, as well as remarkable flexibility and deformability. The maximum tensile strength was 426 kPa, and the maximum elongation at break was 24.7%. When utilized as a wearable electronic device to detect body movements, the porous sponge sensor exhibited rapid and stable sensing responses to external mechanical stimuli, including stretching, compression, and bending. In addition, the incorporation of MPs into the composite endowed it with excellent magnetic sensing capabilities. As the external magnetic field increased from 100 to 240 mT, the maximum electrical response of the SA/CMC composite sensor varied from 20.1% to 50.2%. Moreover, the electrical response of the porous sponge sensor demonstrated reliable stability and reproducibility under periodic external magnetic fields and mechanical stimulation. Consequently, the SA/CMC multiblock foam sensor can function as a bimodal sensor to detect strain and magnetic fields in complex environments. Notably, owing to its favorable electrical response characteristics to strain, it is suitable not only as a wearable electronic device for monitoring human joint motion but also as a finger tactile and joint motion sensor in robotics. Because of its excellent performance, the SA/CMC multiblock foam sensor has the potential to inspire advancements in smart wearable electronics and soft robotics.

## 4. Materials and Methods

### 4.1. Materials and Chemicals

SA (*M*_w_ = 10–15 kDa), CMC (average *M*_w_ = 250,000), GL (AR, relative *M*_w_ = 92.09), and anhydrous CaCl_2_ (AR, relative *M*_w_ = 110.98) were obtained from Shanghai Mai Lin Biochemical Co., Ltd. (Shanghai, China). Micrometer-sized CIPs (≈3.5 μm) and nanoscale Fe_3_O_4_ particles (≈20 nm) were synthesized using chemical coprecipitation [60]. Additionally, bi-dispersed MPs made from GE (1.27 g/mL) and coated with MWCNTs (purity ≥95%) were supplied by Tianjin Hengxing Chemical Reagent Co., Ltd. (Tianjin, China).

### 4.2. Preparation of SA/CMC Porous Foams

Based on our previous study, bidispersed MPs containing CIPs, Fe_3_O_4_, and GE/MWCNTs were prepared [32]. Firstly, SA powders of different qualities were dissolved in 25 mL of distilled water and then magnetically stirred at 60 °C for 30 min. Then, different mass fractions of CMC solutions were added into the above solutions, and stirring was continued at 60 °C for approximately 30 min. Subsequently, 10 mL of CaCl_2_ solution with a concentration of 1 wt% was slowly added to the solution and stirred for another 1 h to obtain a homogeneous SA/CMC polymer solution. After ultrasonic shaking and vacuum drying at 40 °C for 2 h, the SA/CMC gel was gradually formed by eliminating microbubbles. Finally, the polymer gel was frozen at −60 °C for 8 h and dried at 0.1 Pa for 12 h to obtain lightweight, dry magnetic SA/CMC porous foams. The detailed experimental compositions of the SA/CMC porous sponge complexes are listed in Table 2.

### 4.3. Characterization

All measurements were performed at room temperature unless otherwise stated, and the morphology of the SA/CMC porous sponges was characterized using an SEM (Gemini 500, Carl Zeiss AG, Oberkochen, Germany); equipped with EDS. The pore size and its distribution were statistically analyzed by the direct observation of the sections, and the same area and scale as the electron micrographs were selected for the measurements. ImageJ 1.53t software (Wayne Rasband and contributors, National Institutes of Health, Bethesda, MD, USA) was used for the measurements. The results are expressed as the mean ± standard deviation (SD). Characteristic structural peaks were measured by FTIR (Spectrum 100, PerkinElmer, Shelton, CT, USA) in the wavelength range of 4000 to 400 cm^−1^. XPS was performed using a Thermo Escalab 250XI spectrometer (Thermo Fisher Scientific, Waltham, MA, USA). XRD (TTR-III, Rigaku, Tokyo, Japan) was used to examine the crystalline phase characteristics of the porous SA/CMC sponges. The mass–temperature curves of various SA/CMC porous sponges were recorded using a thermogravimetric analyzer (TG; STA 449C, NETZSCH, Selb, Germany).

### 4.4. Performance Testing

The tensile mechanical properties and tensile strain-related electrical responses of the SA/CMC porous sponge samples were tested using a flexible device analysis system (AES-4SD; Beijing Zhongju High-Tech Co., Ltd., Beijing, China). The tensile sample size was 20 mm × 5 mm × 2 mm [GB/T 50081-2019] [61], and the loading speed was set to 4 mm/min. For the water absorption experiments, dried SA/CMC porous sponge samples were dissolved in distilled water at room temperature and cut into 10 mm × 15 mm × 3 mm pieces during the swelling process. After 2 h of swelling, the samples were weighed and the water absorption ratio was obtained using the following equation:

(1) $$R_{w}=\frac{M_{2}-M_{1}}{M_{1}}\times 100\%$$

where $M_{1}$ and $M_{2}$ are the weights of SA/CMC porous sponges in the initial and steady states, respectively. In addition, the sensing signal was characterized using the following equation:

(2) $$\Delta R/R_{0}=\frac{R-R_{0}}{R_{0}}\times 100\%$$

where $R_{0}$ and R are the initial and measured resistances, respectively, and ΔR is the variation in resistance.

## Figures and Tables

**Table 1:** Comparison between SA/CMC sensors and reported dual-modal sensors.

Materials	Stimuli	$\left|∆R/R_{0}\right|$	Advantages	Reference
Lastex yarn (LY)/Ag/PDMS/CIP composite fiber	Strain	~120.0% at 50% strain	Ideal flexibility, large deformation, and appropriate elasticity and sensing properties	[54]
	Magnetic field	~7.6% at 240 mT		
Carbon-fiber aerogel (CFA)/Fe_3_O_4_ silicone composite	Strain	~68% at 20% strain	Highly responsive and wide working frequency	[55]
	Magnetic field	~86% at 150 mT		
Carbon nanotube (CNT)/CIP PDMS sponge	Strain	~82.8% at 60% strain	High flexibility and low density	[56]
	Magnetic field	~3.6% at 144 mT		
Ag/CIP PDMS pillar forest	Strain	~99.6% at 60% strain	Fast response, excellent sensitivity, and high stability.	[57]
	Magnetic field	~0.9% at 170 mT		
CNT/CIP polymeric composite	Strain	~160% at 100% strain	High flexibility, good resistance change, and repeatability	[58]
	Magnetic field	~25% at 340 mT		
AgNW/CIP/flax fiber/MRE fiber	Strain	~19.8% at 16.2° strain	Ideal flexibility, stability, and sensing performance	[59]
	Magnetic field	~11.2% at 60 mT		
SA/CMC porous sponge	Strain	~57.5% at 26% strain	High flexibility, fast response, and high stability	This work
		~115% at 90° strain		
	Magnetic field	~50.2% at 240 mT		

**Table 2:** Detailed experimental composition of SA/CMC porous sponge complexes.

Porous Sponge Types	Sample	Mass Fraction (wt%)		Glycerol (wt%)
		SA	CMC	
SA/CMC Porous Sponges	S1	1	3	2
	S2	2	3	2
	S3	3	3	2
	S4	3	2	2
	S5	3	1	2
	S6	3	2	0
	S7	3	2	4
	S8	3	2	6
SA/CMC Porous Sponges without MPs	S9	3	2	2

## Data Availability

The raw data supporting the conclusions of this article will be made available by the authors upon request.
